# Using a climate attribution statistic to inform judgments about changing fisheries sustainability

**DOI:** 10.1038/s41598-021-03405-6

**Published:** 2021-12-14

**Authors:** Michael A. Litzow, Michael J. Malick, Alisa A. Abookire, Janet Duffy-Anderson, Benjamin J. Laurel, Patrick H. Ressler, Lauren A. Rogers

**Affiliations:** 1 National Oceanic and Atmospheric Administration, Alaska Fisheries Science Center, Kodiak, AK 99615 USA; 2 Northwest Fisheries Science Center, National Oceanic and Atmospheric Administration, Port Orchard, WA 98366 USA; 3 Alaska Coastal Observations and Research, Kodiak, AK 99615 USA; 4 National Oceanic and Atmospheric Administration, Alaska Fisheries Science Center, Seattle, WA 98115 USA; 5 National Oceanic and Atmospheric Administration, Alaska Fisheries Science Center, Hatfield Marine Science Center, Newport, OR 97365 USA

**Keywords:** Climate-change ecology, Ecosystem services, Climate-change impacts

## Abstract

Sustainability—maintaining catches within the historical range of socially and ecologically acceptable values—is key to fisheries success. Climate change may rapidly threaten sustainability, and recognizing these instances is important for effective climate adaptation. Here, we present one approach for evaluating changing sustainability under a changing climate. We use Bayesian regression models to compare fish population processes under historical climate norms and emerging anthropogenic extremes. To define anthropogenic extremes we use the Fraction of Attributable Risk (FAR), which estimates the proportion of risk for extreme ocean temperatures that can be attributed to human influence. We illustrate our approach with estimates of recruitment (production of young fish, a key determinant of sustainability) for two exploited fishes (Pacific cod *Gadus macrocephalus* and walleye pollock *G. chalcogrammus*) in a rapidly warming ecosystem, the Gulf of Alaska. We show that recruitment distributions for both species have shifted towards zero during anthropogenic climate extremes. Predictions based on the projected incidence of anthropogenic temperature extremes indicate that expected recruitment, and therefore fisheries sustainability, is markedly lower in the current climate than during recent decades. Using FAR to analyze changing population processes may help fisheries managers and stakeholders to recognize situations when historical sustainability expectations should be reevaluated.

## Introduction

The signal of anthropogenic climate change is rapidly emerging from the envelope of internal variability in ocean ecosystems around the globe^1–3^. Fisheries are coupled social-ecological systems, and the vulnerability or resilience of fisheries to climate change is determined in part by the adaptive capacity of individual and institutional stakeholders—their ability to minimize harmful outcomes while taking advantage of beneficial outcomes^4,5^. An important component of adaptive capacity is the ability to recognize, attribute, and anticipate change^6,7^. For fisheries stakeholders, a critical step in climate change adaptation is the ability to effectively evaluate the likely impacts on individual fisheries—for instance to evaluate which fisheries are likely to reward the economic investments required to harvest and process fish^8^. This evaluation requires stakeholders to make inferences about how fish populations are likely to fare in ecosystem states that have never been observed^9,10^. Furthermore, the increasing rate of contemporary climate change effects^1–3^ forces stakeholders to make these adaptation decisions based on assessments of likely fisheries outcomes on short time scales (this decade or next decade).

Providing scientific guidance for likely impacts at these shorter time scales presents a daunting technical challenge. Internal variability limits the skill of climate projections over decadal time scales and regional spatial scales^11–14^. Projections of ecological change are also challenged by non-additive interactions between populations and external stressors^15^, driver-response relationships that change over time^16–18^, limited ability to adequately model those complex interactions^19,20^, the propensity for surprising ecological responses to novel combinations of physical conditions^9,21^, and the fundamental limitations for predictability in complex systems^22^. These technical challenges preclude skillful forecasts for the likely responses of individual fish stocks to changing ocean conditions over the short time scale that is immediately relevant to adaptation decisions. Indeed, the magnitude of the challenge for forecasting likely outcomes is highlighted by the observation that determining the cause of fish population collapses is often contentious even after the fact^23–26^.

Recognition and attribution of climate risk also involves barriers of human perception and cognition^4,7^. In particular, evaluation of climate change risks requires individuals and institutions to make the conceptual transition from assessing likely outcomes for exploited populations based on historical experience (backward-looking perspective), to assessments based on the assumption that current climate trends will continue (forward-looking perspective)^10^. The backward-looking perspective is a natural mode of adaptive human behavior since the range of conditions observed in the past was long the best guide for what might be experienced in the future. However, a backward-looking perspective predisposes resource-dependent communities to vulnerability in the face of strong environmental trends^5^. But the transition away from the traditional backward-looking perspective is challenging for individuals and institutions^10^, and the cognitive barriers involved may be accentuated by the complexity inherent in scientific advice concerning likely fisheries outcomes^7^.

Given this set of conditions—rapid emergence of climate change effects, technical challenges for evaluating likely this decade or next decade fisheries impacts, and cognitive barriers to adopting forward-looking assessments of climate risk—there is a need for new approaches for guiding adaptation decisions for fisheries in rapidly changing ecosystems. Here, we present one such approach, combining an empirical evaluation of observed population responses to anthropogenic climate extremes as they emerge in an ecosystem, coupled with inferences on likely changes in probability distributions based on hindcasts and decadal-scale forecasts of the incidence of anthropogenic extremes.

Our approach uses a climate attribution statistic (Fraction of Attributable Risk; FAR^27–29^) to assess observed ocean temperature anomalies along a gradient of likelihood in the absence or presence of anthropogenic radiative forcing. FAR values for primary climate variables such as temperature or precipitation are calculated as 1—preindustrial probability/current probability^27,28^, so FAR estimates the proportion of risk for a particular climate event that can be attributed to human influences. Positive values of FAR can also be thought of as the likelihood that a given anomaly lies outside the envelope of preindustrial variability. We use observed covariance between fish population processes and FAR values to compare population responses to conditions typical of historical climate (low FAR) with conditions outside the envelope of expected preindustrial conditions (high FAR). Projected FAR values from Coupled Model Intercomparison Project Phase 5 (CMIP5) climate models run under Representative Concentration Pathway (RCP) 8.5 (the current emissions trajectory^30^) are then used to generate forward-looking predictions of likely population trends for comparison with backward-looking predictions based on historical FAR values.

**Figure 1:**
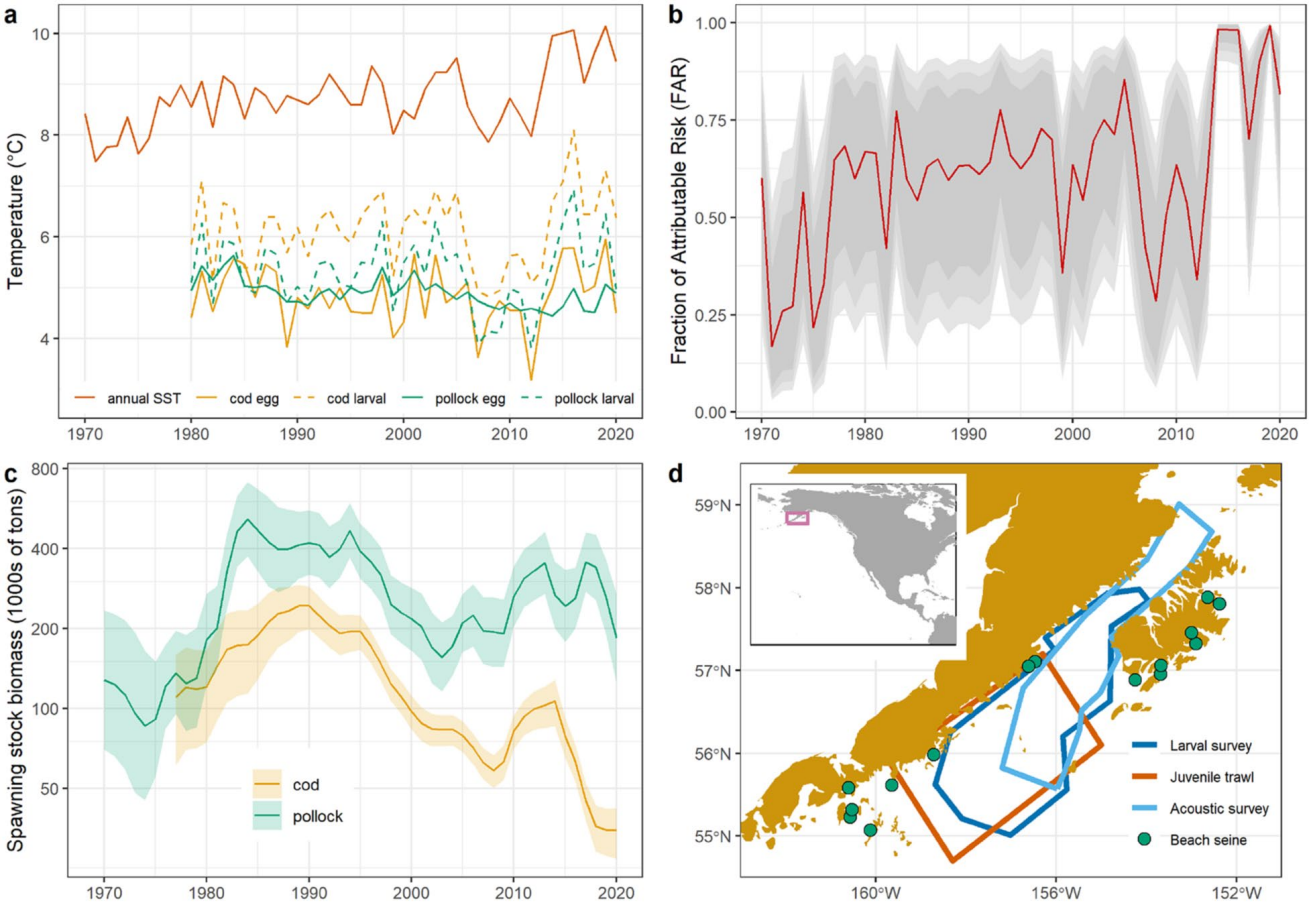
Study system: the Gulf of Alaska Pacific cod and walleye pollock fisheries. (**a**) Temperature time series for annual Gulf of Alaska sea surface temperature (SST), and specific seasons and depths for early life history stages (cod egg = January–April 105 m, cod larval = April–June 15 m, pollock egg = March–May 262 m, pollock larval = March–April 65 m and May–June 25 m). (**b**) Fraction of attributable risk (FAR; 1—preindustrial probability/current probability) for annual Gulf of Alaska sea surface temperature, 1970–2020, mean and 80%/90%/95% CI from Bayesian model comparing ERSSTv5 observations with simulated preindustrial values from five CMIP5 models. (**c**) Estimated spawning stock biomass from age-structured population models^31,32^ with 95% CI (for pollock) and ± 2SD (for cod). Note log scale on y-axis. (**d**) Study area, with locations for field data collection. Inset shows study area (magenta box). Map created in R 4.0.2^69^, http://www.r-project.org/.

We illustrate our approach with populations of two fishes in the Gulf of Alaska: Pacific cod (*Gadus macrocephalus*) and walleye pollock (*G. chalcogrammus*). These populations support economically important fisheries (combined 2015 first-wholesale value $ 207 million USD^31,32^) in a rapidly warming ecosystem. Annual sea surface temperature (SST) values in the Gulf of Alaska exceeded previous maxima for four of seven years during 2014–2020^33^, and these temperature extremes have propagated to depth (> 200 m) over the continental shelf^29^ (Fig. 1a). Comparisons with downscaled CMIP5 preindustrial simulations have indicated that the magnitude and duration of these temperature anomalies would be impossible in the absence of anthropogenic radiative forcing (i.e., FAR = 1^29,33^; Fig. 1b).

Our analysis focuses on recruitment (production of young fish), a key process regulating the size of marine populations^34^. One of our study populations, Pacific cod (hereafter, “cod”), collapsed in 2015–2018 (Fig. 1c), apparently due to the impacts of early life history thermal stress combined with increased metabolic demands and insufficient prey resources for older cohorts^35,36^, though synergistic fishing effects cannot be ruled out^37,38^. The central adaptation question for stakeholders in this fishery is the likelihood for the stock to recover (i.e., to restore catches to the range of historical experience). Recruitment in this species is directly limited by warming, as egg survival peaks in a narrow range of optimal temperatures (≈ 4–5 °C^39^). Walleye pollock (hereafter, “pollock”) have not collapsed, and still support a viable fishery (Fig. 1c). As with many exploited stocks, age structure in pollock is truncated, such that population dynamics are dominated by recruitment^38^. The relevant adaptation question for stakeholders in this fishery is how long recruitment will be adequate to support a sustainable fishery as the climate changes (i.e., continue to produce catches within the range of historical experience). Pollock eggs survive at a wider temperature range (≈ 0–8 °C^40^), so direct warming effects on recruitment are expected to be weaker for this species. However, extreme values in other climate variables, such as salinity, have resulted in reduced recruitment during recent climate anomalies^41^.

We show that anthropogenic temperature extremes are associated with unprecedented, low recruitment probability distributions for both cod and pollock, although the mechanisms involved appear to differ between species. Predictions based on near-term CMIP5 projections indicate that expected recruitment in the 2020s is markedly reduced compared to historical conditions. This approach provides stakeholders with an explicit evaluation of backward-looking and forward-looking perspectives for answering adaptation questions as the ecosystem transitions away from the conditions that have sustained fisheries in the past.

## Results

Our analysis addresses three objectives: (1) we use FAR values for annual Gulf of Alaska SST anomalies to estimate the probability that observed climate conditions fall outside the range of natural (preindustrial) variability; (2) for both species, field data and population model estimates of recruitment (defined as abundance at age-0) are then used to evaluate the response of recruitment to novel anthropogenic conditions within the observational record; and (3) Bayesian models predicting recruitment given historical and projected FAR values are then used to compare expected recruitment distributions under historical and forward-looking perspectives. Additionally, we relate observed recruitment to temperature estimates from the egg and larval periods to determine the degree to which recruitment variability can be directly tied to temperature effects.

**Figure 2:**
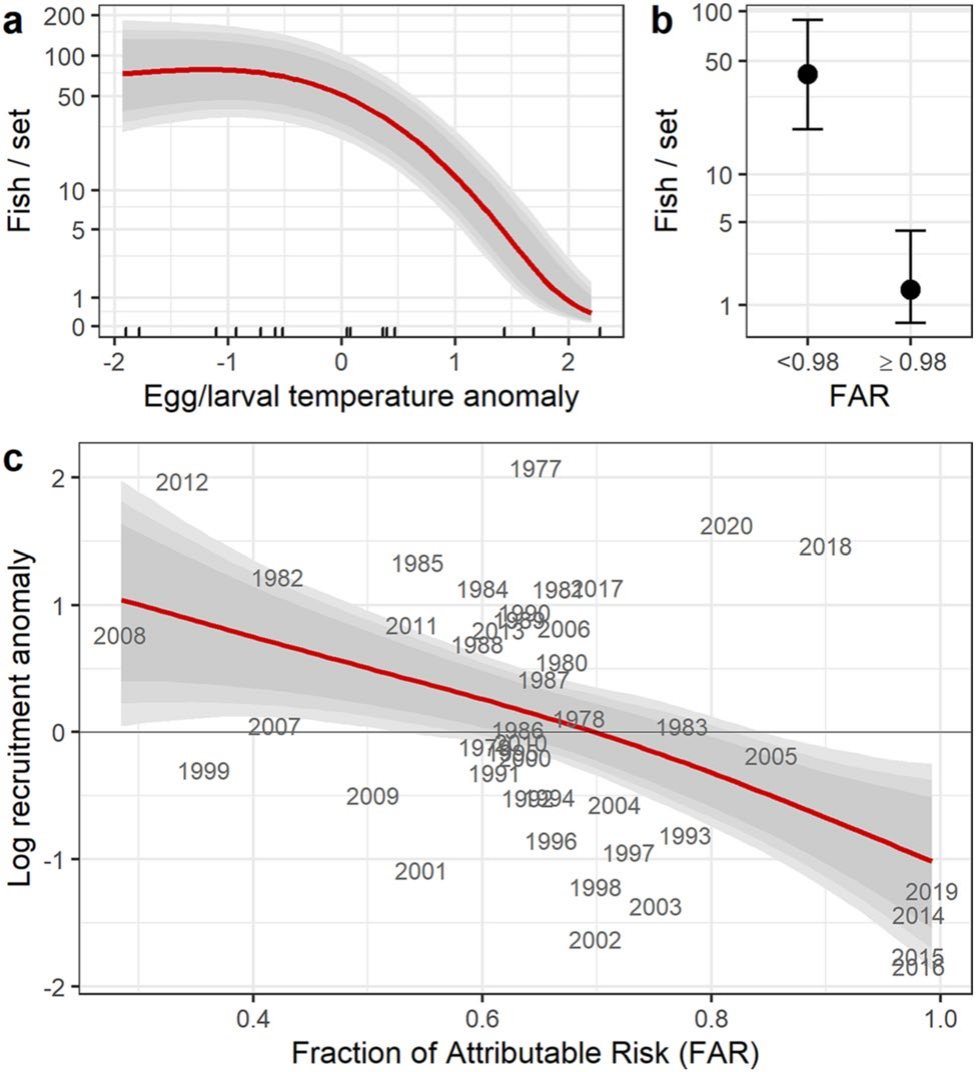
Anthropogenic temperature extremes and Pacific cod recruitment. (**a**) Age-0 recruitment from beach seine data as a function of egg/larval temperature. Grey ribbons indicate 80%/90%/95% CI. (**b**) Age-0 recruitment from beach seine data as a function of the Fraction of Attributable Risk (FAR) for Gulf of Alaska SST anomalies (error bars = 95% CI). (**c**) Estimated cod recruitment from stock assessment model (1977–2016; 2017–2020 values derived from beach seines) as a function of FAR: predicted values and 80%/90%/95% CI. Recruitment log-transformed and scaled as zero mean, unit variance.

Field observations of cod recruitment come from a 15-year time series (2006–2020) of beach seines that sample cohorts as newly settled age-0 fish (Fig. 1d). We compared a set of alternative hierarchical Bayesian regression models to predict the effect of temperature during the egg and larval phases on age-0 cod recruitment. The best model also controlled for spawning stock biomass, date of sampling, and bay and site effects. This model predicts a two order of magnitude decline in expected recruitment with observed warming (Fig. 2a, Bayesian R^2^ = 0.37 [95% CI 0.22–0.53], see Table 1 for detailed results on model selection and SI for parameter estimates). Beach seines were conducted during four years with FAR values ≥ 0.98, indicating SST anomalies that would have been highly unlikely in the preindustrial ocean. A Bayesian model treating FAR values as a categorical covariate (above or below 0.98) shows that recruitment failed (that is, declined by an order of magnitude from the long-term mean) in all four of these years (estimated recruitment of 42 fish set^−1^ for FAR < 0.98 [95% CI 19–89 fish set^−1^], and 2 fish set^−1^ at FAR ≥ 0.98 [95% CI 0–4 fish set^−1^]; Bayesian R^2^ = 0.34 [95% CI 0.20–0.15], Fig. 2b). This result provides the first indication that cod recruitment fails at temperature extremes that are highly likely to fall outside the envelope of preindustrial conditions.

**Table 1:** Model selection results.

Model set	Response variable	Notes	Covariates	LOOIC	Δ-LOOIC
M1	Cod seine abundance	Figure 2a	DOY + bay/site + temp + SSB	7770.98	0.00
M1			DOY + bay/site + temp	7815.16	44.18
M1			DOY + bay/site	7962.38	191.39
M1			DOY + bay	8083.84	312.86
M2	Cod annual seine abundance	For comparing to stock assessment model	Year + DOY + bay/site	7519.75	0.00
M2			Year + DOY + bay	7708.33	188.58
M3	Cod stock assessment model recruitment	To estimate 2017–2020 values in Fig. 2c	Seine	11.98	0.00
M3			Seine + SSB	17.82	5.84
M4	Pollock DFA trend	Figure 4a	FAR	91.10	0.00
M4			FAR + SSB	93.01	1.91
M4			Larval temp	93.52	2.42
M4			SSB + larval temp	95.56	4.46
M4			Egg/larval temp	95.65	4.55
M4			SSB + egg/larval temp	96.32	5.22
M5	Pollock model recruitment estimate	Figure 4b	FAR	127.66	0.00
M5			FAR + SSB	129.91	2.25

*DOY* day of year, *SSB* spawning stock biomass, *temp* egg/larval temperature, *FAR* fraction of attributable risk, *LOOIC* leave one out information criterion, *Δ-LOOIC* LOOIC difference from best model.

As a second source of cod recruitment information, we consider the time series of estimated recruitment from the age-structured stock assessment model, which begins in 1977^32^. Values for 2017–2020 are poorly supported by data in the model, so we estimated these values from the beach seine data (see “Methods” for details). This analysis uses log recruitment anomalies, which have been centered on zero and scaled to unit variance. Bayesian regression shows a nearly linear, declining response of log recruitment anomalies in this time series to estimated FAR values within the observed record (Bayesian R^2^ = 0.22 [95% CI 0.04–0.39]; Fig. 2c). This result includes recruitment failure (defined as log anomaly < − 1) for FAR values ≥ 0.98 during 2014–2016, providing independent confirmation of recruitment failure at high FAR values (i.e., the recruitment estimates for those years are not informed by the seine data). These results lead us to conclude that, within the observational record, cod recruitment has failed whenever ocean temperatures have exceeded preindustrial values, as indicated by FAR ≥ 0.98.

**Figure 3:**
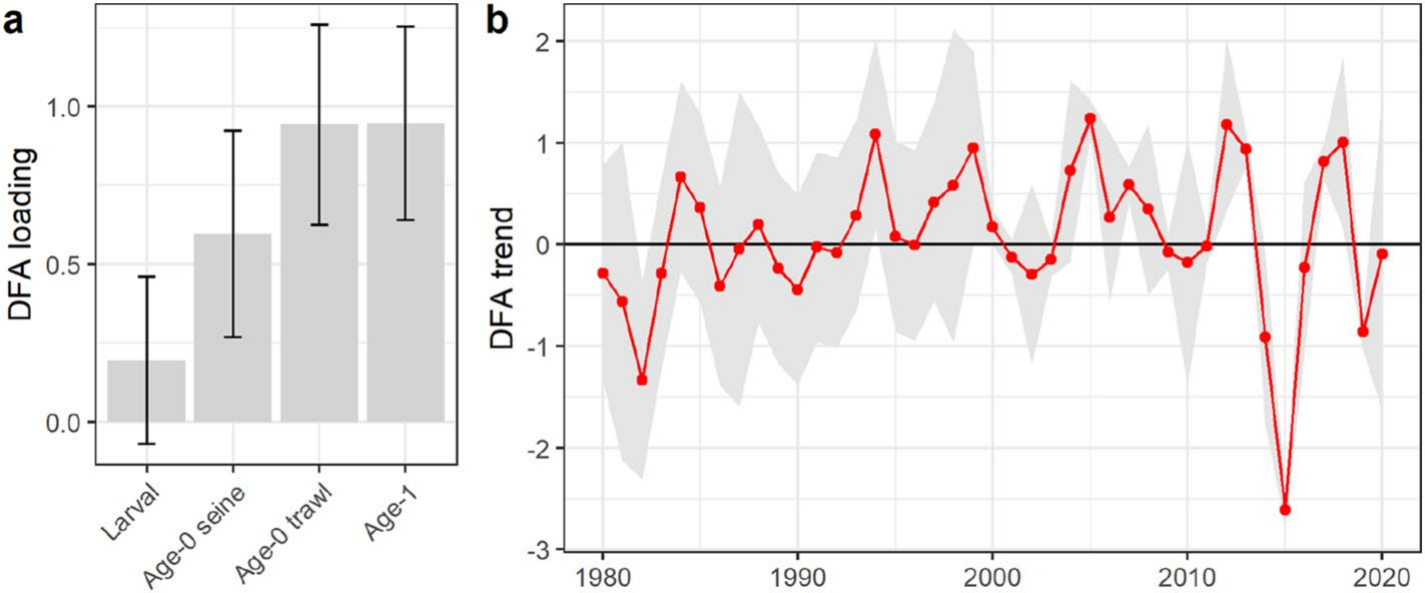
Dynamic Factor Analysis (DFA) results for shared trend in field observations of pollock recruitment, 1981–2020. (**a**) Time series loadings and 95% CI. (**b**) Shared trend in variability, with 95% CI. Positive values of the DFA trend in (**b**) can be interpreted as an increase in an unobserved process (e.g. pollock recruitment) that shows a positive relationship with the three time series in (**a**) with CIs for loadings that do not include zero.

Results for pollock were less consistent, with little evidence for a direct effect of pre-settlement temperatures on recruitment. We used four data sets to estimate age-0 pollock recruitment: a pelagic larval survey, beach seines, a juvenile trawl survey, and an acoustic trawl survey of age-1 abundance, which we lagged by one year for analysis (Fig. 1c, see “Methods” for details). The four time series were log transformed and combined using Dynamic Factor Analysis (DFA) to estimate a single shared trend in log age-0 pollock abundance for 1981–2020 (see “Methods” for details). The DFA model for pollock field observations showed positive loadings on age-0 abundance from seines and trawls and age-1 abundance from the acoustic survey, but a loading for larval surveys that could not be distinguished from zero (Fig. 3a). This result indicates a shared trend in recruitment that is tracked by age-0 and age-1 surveys, but less well by larval data. The shared trend indicated a series of low recruitment years since the onset of extreme temperatures in 2014 (Fig. 3b). However, Bayesian regression models invoking temperature during the egg and larval phases showed estimated temperature effects with 95% posterior intervals that spanned zero, leading to the conclusion that available data are not consistent with direct temperature effects on recruitment (full results in SI).

**Figure 4:**
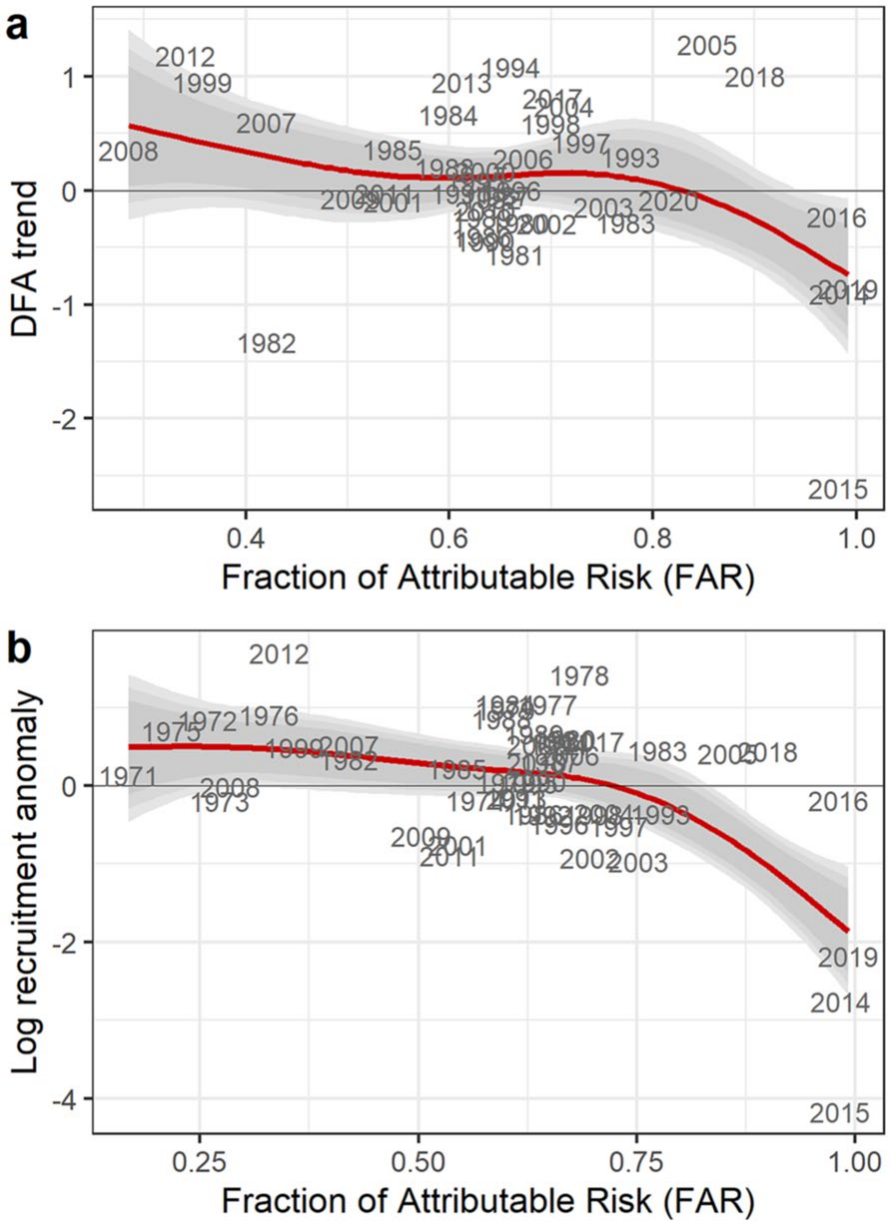
Anthropogenic temperature extremes and walleye pollock recruitment. (**a**) Dynamic Factor Analysis (DFA) shared trend of four field time series for age-0 recruitment as a function of FAR values for Gulf of Alaska sea surface temperature anomalies. (**b**) Estimated age-0 pollock recruitment from stock assessment model (1970–2019) as a function of FAR: predicted values and 80%/90%/95% CI. Recruitment log-transformed and scaled as zero mean, unit variance.

Conversely, the best Bayesian regression model invoking FAR for Gulf of Alaska SST did show a negative effect of extreme FAR values on field observations of age-0 pollock abundance (Bayesian R^2^ = 0.20 [95% CI 0.03–0.39]; Fig. 4a). As with cod, we evaluated the evidence of reduced recruitment under anthropogenic temperature extremes with an additional analysis using stock assessment model estimates of recruitment for the years 1970–2019. A Bayesian regression model indicates a threshold response to anthropogenic warming, with a greatly increased chance of recruitment failure for FAR ≥ 0.98 (Bayesian R^2^ = 0.35 [95% CI 0.16–0.51]; Fig. 4b).

**Figure 5:**
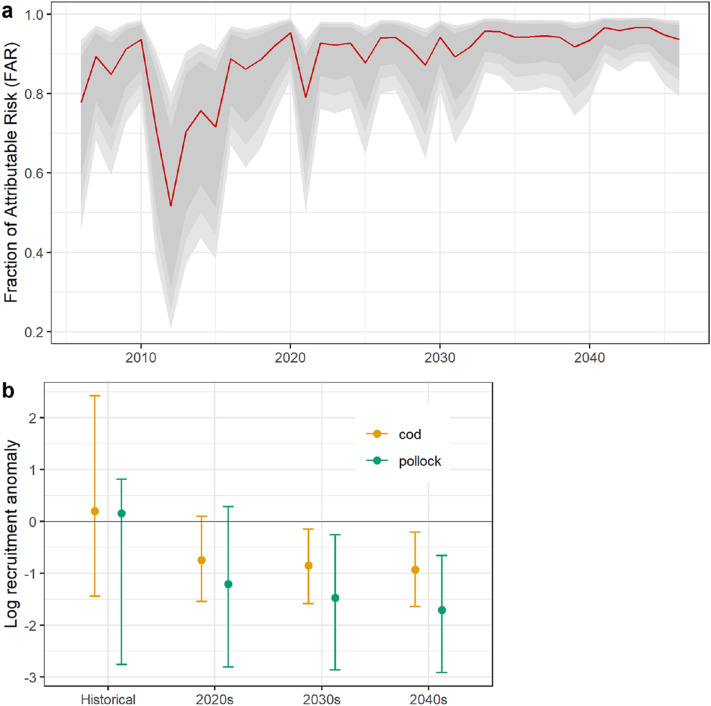
Historical and forward-looking predictions of cod and pollock recruitment. (**a**) Bayesian time series modeling of CMIP5 projected FAR values under RCP8.5, 2006–2046. Red line indicates predicted FAR value, with grey ribbons indicating 80/90/95% CI due to model uncertainty. (**b**) Predicted cod and pollock recruitment based on historical observed FAR values (1970–2019 for pollock, 1977–2019 for cod) and current/future decades under CMIP5 RCP8.5 FAR projections: median values with 95% CI.

To compare judgments concerning cod and pollock fisheries sustainability based on backward-looking and forward-looking perspectives, we compare predicted recruitment under historical and projected FAR values for the Gulf of Alaska. The historical time period begins with the start of recruitment estimates from stock assessment models (1977 for cod, 1970 for pollock) and ends in 2019; FAR values for this period are calculated with observed SST values. Output from CMIP5 RCP8.5 experiments are available for 2006–2046^29^ (see “Methods”). FAR values for projecting cod and pollock recruitment distributions are calculated using these CMIP5 projections for 2020–2046. We also plot the Bayesian FAR estimates for the full 2006–2046 RCP8.5 projection time series in order to illustrate the transition away from the envelope of natural variability. Median projected FAR values are below 0.9 for much of the 2010s, consistent with the view that 2014–2016 temperature anomalies were the result of both anthropogenic forcing and extreme internal variability^29^. During the 2020s, median FAR values commonly exceed 0.9 and become fixed above 0.95 in the 2030s and 2040s (Fig. 5a), indicating the emergence of an anthropogenic climate state, with temperatures that were previously novel extremes rapidly becoming the mean state. Projected recruitment values for both species track the emergence of this novel climate state, with declines in both the central tendency and variance of recruitment in the 2020s compared with historical projections, and continuing declines in the 2030s and 2040s (Fig. 5b). The projected declines for the 2020s correspond to a 38% (88%) decline in median recruitment for cod (pollock) compared with historical estimates (original units, not log-transformed).

## Discussion

The high FAR values observed for SST (FAR ≈ 1) indicate anomalies that cannot be explained without anthropogenic radiative forcing^29^, and the appearance of these anomalies signals the transition away from conditions captured by historical experience in the Gulf of Alaska ecosystem^10^. This transition subjects fisheries to increased volatility and the risk of unpleasant surprises^10,42^, such as the Gulf of Alaska cod collapse^35^. There is a need for the fisheries science community to develop new tools for providing stakeholder guidance concerning likely fisheries outcomes under these conditions. When compared to other possible covariates, such as temperature^39^ or a heatwave index^35^, climate attribution statistics have the advantage of making it clear that human-induced warming is a driver of population variability in the system. This attribution is an important step for successful adaptation^6,7^. Here, we evaluate the potential for changing sustainability in individual fisheries with empirical evaluations of two questions: how has the population behaved in the past when the climate has exceeded preindustrial limits, and what are the distributions of climate events outside the preindustrial envelope historically and in short-term future projections? Formal attribution of observed climate impacts on individual marine populations (i.e., estimating the contribution of anthropogenic radiative forcing relative to other causes) may be an intractable problem because of the current barriers to effective modeling of complex ecological relationships^15^. This sort of formal attribution is not our goal in using a climate attribution statistic as a covariate in the present study. Rather, our goal is to summarize empirical responses to anthropogenic temperature extremes to explicitly frame the differences between backward-looking and forward-looking assessments of likely population stability and fisheries sustainability. To our knowledge, FAR values have not previously been used in this kind of adaptation context. The FAR approach was developed for attribution of extreme weather events^27,43^, and remains the most common attribution approach in that context^44^. FAR has also been used to attribute the risk of an extreme wildfire season^45^ and the economic damages of extreme weather events^46,47^, and a closely related approach has been used to attribute greening trends in global terrestrial vegetation^48^. Our study presents a strictly probabilistic approach to estimating changes in a coupled social-ecological system exposed to climate events beyond the envelope of preindustrial variability. While it suffers from the limitations of any statistical modeling based on association among variables, it also avoids the complexities of a “case study” approach, whereby the different causal factors and mechanistic linkages are examined in a forensic approach to understand anthropogenic contributions to a given event^44,49^. Different attribution approaches are valid in different contexts^44^, and our hope is that our application of attribution tools in this context will combine empirical rigor with simplicity in a way that may guide effective adaptation decisions^7^.

For Pacific cod, a direct effect of temperatures on hatch success is well established^39^ and this direct temperature effect is supported by our analysis (Fig. 2a). This mechanism of thermal sensitivity increases our confidence in the forward-looking assessment that successful recruitment for this species is increasingly unlikely. Conversely, pollock recruitment was not directly tied to temperature, but it did show a strong negative response to annual SST values that were likely outside the envelope of preindustrial variability, as indicated by high FAR values (Fig. 4). This combination of results at first seems counter-intuitive. However, extreme temperature anomalies in the Gulf of Alaska have co-occurred with unprecedented conditions in a range of other climate variables relevant to early life history pollock, including low salinity throughout the water column^41^, reduced wind mixing^50^, and reduced alongshore transport^50^. Further, direct temperature effects on early life history stages may depend on prey timing and abundance^41,51^. The separate effects of these collinear mechanisms, operating under novel patterns of covariance among climate drivers^50^, are not currently understood in a predictive sense that would support adaptation decisions.

Our analysis frames climate-recruitment relationships in a way that provides empirically rigorous, intuitive scientific advice for stakeholders: cod and pollock have experienced poor recruitment when Gulf of Alaska temperatures exceeded the limits of preindustrial variability in the past, and the projected incidence of further anthropogenic climate extremes suggests that the probability of successful recruitment has declined markedly compared to historical values. The 38–88% decline in median, non-log transformed recruitment projected for the 2020s would likely be sufficient to ensure a reduced likelihood of recovery (for cod) or increased likelihood of population declines (pollock). These projections suggest that sustainable fisheries for these populations are increasingly unlikely in the contemporary Gulf of Alaska. However, observed climate-biology relationships are prone to failure when deployed out of sample^16^. In particular, the effects of novel climate conditions on recruitment may be transient^52^, and fish populations may adapt or acclimate to warming temperatures^53^. Better understanding of mechanistic drivers remains an important research need in order to reduce these uncertainties.

Modeling uncertainties are also important considerations for our study. While the five CMIP5 models used in this analysis were selected based on their simulations of historical temperature, precipitation, and sea level pressure over Alaska^54^, our analysis remains vulnerable to error in the regional CMIP projections used to calculate FAR^13,14^. The effect of this error may be accentuated because of the different mix of data and model outputs used to estimate historical FAR (preindustrial simulations and observed data) and projected FAR (preindustrial simulations and RCP8.5 projections, see “Methods”)^55^. In addition, the statistical downscaling method used to process CMIP5 outputs is vulnerable to changes in model bias over time^54^.

These uncertainties suggest caution when evaluating our conclusions concerning emerging losses of sustainability with anthropogenic climate extremes. However, the magnitude of projected change in Gulf of Alaska climate also highlights the potential shortcomings of retaining the historical perspective for guiding adaptation decisions. North Pacific temperature anomalies during 2014–2018 peaked at ≈ 4.5 SD above recent climatology^56^, and CMIP5 simulations estimate that a basin-scale event of this magnitude was expected less than once every 10,000 years in the preindustrial ocean^2,56^. Projections under RCP8.5 conditions estimate an expected return time for a similar-magnitude event to be every ≈ 30 years with a 1 °C anthropogenic increase to mean global surface temperature (i.e., 2020 conditions), and every ≈ 10 years with 1.5 °C of global warming (≈ 2040s conditions)^2,57^. Similar patterns are seen at the regional scale in the downscaled CMIP5 projections used in the current study^29^.

We note that the largest part of the projected decline in forward-looking assessments compared with backward-looking assessments has already occurred by the 2020s, with marginal declines in subsequent decades (Fig. 5). This pattern reflects the emergence of FAR values > 0.98 with the onset of anthropogenic warming in the system. Because FAR values cannot exceed 1, our approach is useful for assessing population responses to anthropogenic conditions as they emerge, but it does not provide insight into the fisheries impacts of continued warming within the anthropogenic state.

Given the relationships between anthropogenic temperature anomalies and recruitment failure for these populations, what are the implications for climate adaptation decisions by stakeholders? For harvesters and processors, taking on a forward-looking assessment of recruitment probabilities results in very different conclusions concerning the likely risk versus return for economic investments to participate in these fisheries^10^. For fisheries managers, the forward-looking perspective highlights the need to abandon assumptions of long-term average conditions that describe population processes. Slow adaptation by fisheries managers that stems from slow recognition of changing population dynamics predisposes fisheries to collapse^23,58^. Preserving important fisheries with declining sustainability characteristics for as long as possible to give new fisheries the opportunity to develop is also an important step in climate adaptation^6^. Important moves towards developing more forward-looking perspectives for Gulf of Alaska fisheries have already been developed. These include a standardized process for evaluating climate risk that is not captured by stock assessment models^59^, and the inclusion of climate covariates in stock assessments^32^.

The differences in expected recruitment under historical and forward-looking perspectives (Fig. 5) are statements of probability (model posteriors), and the 95% credible intervals for recruitment in the 2020s include the historical mean values. The potential for successful recruitment even under increasing chances of extreme anthropogenic temperatures is underscored by observations of successful age-0 recruitment for both populations in 2018 and 2020. In addition, temperature in the Gulf of Alaska exhibits high levels of interannual autocorrelation^60^, which leaves open the chance of a multi-year cooling event that would offer reprieve from the warming trend. However, the strong expected climate trend in this system is likely to overwhelm any beneficial effects due to climate variability^10^, which suggests that any such reprieve would be best used to prepare for the negative outcomes suggested by the forward-looking perspective.

## Methods

### Field sampling and stock assessment models

Beach seines were conducted with a negatively buoyant, 36 m long seine, with wings 1 m deep at the ends expanding to 2.25 m in the middle, 13 mm mesh in the wings and 5 mm delta mesh in the cod end bag. Seine wings were attached to 25 m ropes for deployment and retrieval from shore, and effective sampling area was ~ 900 m^2^ of bottom habitat. Sampling was conducted during July and August at 95 sites in 15 bays, with each site sampled 1–4 times per year (*n* = 1,145 sets). The two easternmost bays were sampled each year during 2006–2020, with at least two sampling visits per year. The remainder of bays were sampled during 2018–2020. A Bayesian regression model invoking bay, site, seasonal timing of sampling, and year effects shows a strong correspondence with cod recruitment estimates from the stock assessment model for 2006–2016. The model further indicates that bay effects were weak compared to interannual differences in abundance.

While age-0 cod preferentially occupy nearshore habitats that are well sampled by beach seines, we were concerned that age-0 pollock are more likely to be offshore and thus poorly sampled by seines. We therefore made the a priori decision to limit analysis of seine pollock data to six bays where pollock were most commonly captured. However, Bayesian regression model estimates of annual age-0 abundance from this restricted set of bays were nearly identical to annual estimates from the full data set (r > 0.99).

Pollock larvae were sampled during 1981–2019, with six missing years, and juvenile pollock trawl surveys were conducted during 2000–2019, with nine missing years. Larvae were sampled over a fixed area (Fig. 1d) during mid-May–early June using oblique tows from 10 m off bottom (or 100 m depth maximum) to the surface using a 60 cm diameter bongo net (333 or 505 µm mesh). Calibrated flowmeters in each net estimated the volume filtered. A time series index of larval pollock abundance was calculated as the area-weighted mean catch 10 m^−2^^41^. Trawl surveys for age-0 pollock were conducted in a fixed area (Fig. 1d) in August–September. Samples were collected using a midwater trawl fished with 1.5 × 2.1 m steel V-doors (566 kg each) and equipped with a 3 mm codend liner. The trawl was fished obliquely through the water column at a ship speed of 2.5 to 3.0 knots and a wire retrieval rate of 10 m min^–1^. A time series of age-0 pollock abundance was developed by calculating an area-weighted mean catch m^−2^ in each year, using the same methodology as for the larval index.

Acoustic trawl surveys of pre-spawning pollock were conducted during 1981–2020 with four missing years^61^. Acoustic backscatter at 38 kHz classified as walleye pollock by trained analysts is sampled with large midwater and bottom trawls to determine species and size composition. Abundance and biomass of pollock at age are estimated from 16 m below the sea surface to 0.5 m above the seafloor. The age-1 index from this time series was lagged one year for our analysis.

Finally, estimates of spawning stock biomass and recruitment were obtained from the most recent versions of age-structured assessment models used in management of the two stocks^31,32^. Recruitment at age-0 is from lagged estimates of abundance at age-1 for pollock, and age-3 for cod.

### Climate data and attribution statistics

We use the average of Global Ocean Data Assimilation System monthly temperature anomalies for the cod and pollock egg and larval phases to evaluate the direct effects of pre-settlement temperatures on age-0 recruitment. Cod (pollock) egg temperatures were calculated as January-April 105 m (March–May 262 m) means, and cod (pollock) larval temperatures were calculated as April-June 15 m (March–April 65 m and May–June 25 m) means (see SI for details).

Calculation of FAR values follows the approach suggested in Stone et al. (ref^62^). The analysis focuses on two vulnerabilities: the probability of reduced recruitment with extreme values in temperature and collinear climate variables^41,50^, and the vulnerability of a historical perspective concerning likely recruitment probabilities. FAR calculations are framed on annual, regional-scale SST values. This spatially-temporally broad SST selection, which was based on the availability of prior attribution studies^29,33^, may avoid selection bias from a more spatially-temporally focused temperature variable known to be associated with the cod collapse^35^ at the potential cost of making our conclusions concerning FAR values conservative^62^. Analysis was conditioned on previously-published downscaled SST outputs from five CMIP5 models showing good predictive skill for Alaskan climate variability (CCSM4, GFDL.CM3, GISS.E2.R, IPSL.CM5A.LR, MRI.CGCM3)^29,54^, and was focused on the probability of SST anomalies with anthropogenic forcing, rather than the magnitude of anomalies^62^. Downscaled outputs were made available as a single 60-year realization under preindustrial conditions and a single 60-year realization from the RCP8.5 simulation for each model. Observational SST anomalies are calculated from ERSSTv5^33^. The distribution of observed SST anomalies was calculated for the 1961–2020 period, scaled by the 1900–2020 SD (i.e., SST anomaly = [annual value—1961–2020 mean] / 1900–2020 SD)^33^. FAR values for the historical period were calculated as 1—preindustrial probability/observed probability, and FAR for projected conditions were calculated as 1—preindustrial probability/RCP8.5 probability, with probability in all cases being measured as the proportion of anomalies greater than or equal to the anomaly in question. We then used a Bayesian regression model with a beta distribution to estimate the mean FAR value for each year. The model included fixed-year effects and accounted for uncertainty due to the spread in FAR values among the CMIP5 models by treating model identity as a random effect. Because the beta distribution requires values between 0 and 1, we reset FAR values of 1 to 0.9999 prior to analysis.

### Analysis

Dynamic Factor Analysis (DFA) is a dimensional reduction technique that is designed for time series data and robust to missing values^63^. We estimated field values of pollock recruitment with a DFA model fit in the R package MARSS, using an error structure with different time series variances and no covariance^64^.

We used a Bayesian zero-inflated negative binomial (ZINB) model to estimate the relationship between seine catch per unit effort for cod and mean temperature anomaly during the egg/larval phase^65,66^. In addition to temperature anomaly, continuous covariates included day of the year of sampling and spawning stock biomass. To account for differences across sampling locations we also included random terms for sampling bay and site within bay. The continuous and random terms were included in both the zero-inflated (binomial) and abundance (count) parts of the model. We used a logit link function for the zero-inflated component and log links for the abundance component and shape parameter. Estimates of the effect of FAR values on cod abundance in seines were made with a Bayesian Gaussian model invoking two FAR levels as a categorical covariate (high/low, corresponding to FAR above or below 0.98 in the observational time series). The four years with FAR ≥ 0.98 (2014–2016, 2019) also showed high levels of model agreement in FAR estimates (95% credible intervals indicating at least 89% of the risk was human induced; Fig. 1b). We judged that these four years, with precise estimates of extremely high FAR values, served as a suitable set of clearly anthropogenic extremes to compare with other years characterized by lower FAR and greater model uncertainty (Fig. 1b). We did not evaluate the effect of the choice of the 0.98 threshold on these results.

Stock assessment model estimates for cod recruitment during 2017–2020 are poorly informed in the model (i.e., those year classes have not yet recruited to adult surveys or the fishery). We used a two-step approach to estimate recruitment for those years. First, we estimated annual abundance for beach seines (2006–2020) using a ZINB model that included a day of year effect and random effects for sampling location. Second, we predicted recruitment for 2017–2020 using a linear regression model that was fit using only the years of overlap between assessment recruitment (response variable) and model estimated CPUE (dependent variable; 2006–2017). We then fit a Bayesian additive model to estimate the relationship between recruitment (1977–2017 from stock assessment; 2018–2020 from seine estimates) and FAR. We also considered candidate models invoking spawning stock biomass to account for density-dependent effects on recruitment. We used thin plate regression splines to account for high degrees of freedom relationships between recruitment and the covariates FAR, spawning stock biomass, and sampling day of year^67^.

A similar approach was used for pollock, where Bayesian additive models were fit to both the DFA trend for field data and stock assessment model estimates in order to estimate the effect of FAR on recruitment. The age-1 abundance data included in the pollock DFA are a data input to the stock assessment model, so the two analyses of FAR effects on pollock recruitment are not independent. However, similar results were obtained with a DFA on field data that excluded the age-1 time series.

While plots of observed recruitment-FAR relationships (Figs. 2, 4) do not account for uncertainty in FAR values, we obtained qualitatively similar results using models that account for uncertainty in FAR, which we characterized as the standard error of annual FAR estimates from Bayesian regression models. Because we wanted to account for model uncertainty in FAR values when comparing estimates of historical and forward-looking recruitment distributions, we used the models that accounted for FAR uncertainty when making those estimates (Fig. 5).

Each regression model was fit using Stan 2.21.0^68^, R 4.0.2^69^ and the brms package^70^. All estimated parameters had a potential scale reduction factor ($\widehat{R}$) less than 1.05, an effective sample size of at least 1000, and no divergent transitions were observed. We also assessed chain convergence and model fits using graphical methods (e.g., trace-plots) and posterior predictive checks^71^. To avoid overfitting, smooths in thin plate regression splines were limited to 3 or 5 basis functions, depending on the particular covariate and duration of available data. Model selection was made by minimizing the Leave One Out Information Criterion. Details on model parameter estimates are presented in the SI.

### Ethics statement

All field sampling was conducted under Alaska state and U.S. federal permits issued to participating organizations. No live animal experiments were conducted during this study. The biological data used in this study were collected as a part of routine population monitoring to inform fisheries management.

## Supplementary Information

Supplementary Information.

## Data Availability

All data and code necessary to replicate the findings of this study are available in the fish-FAR repository with the identifier https://github.com/mikelitzow/fish-FAR (permanent repository on Zenodo, https://doi.org/10.5281/zenodo.5535032). Larval pollock data are also accessible at: https://access.afsc.noaa.gov/ichthyo/index.php.
