# Supplementary material for: Using a climate attribution statistic to inform judgments about changing fisheries sustainability
Source: Sci Rep. 2021 Dec 14;11:23924. doi: 10.1038/s41598-021-03405-6 (PMC8671533; doi:10.1038/s41598-021-03405-6)
Supplement: Supplementary file 1 — Supplementary Information. [file 41598_2021_3405_MOESM1_ESM.pdf]

Supplemental Information

Using a climate attribution statistic to inform judgments about changing fisheries sustainability

Michael A. Litzow, Michael J. Malick, Alisa A. Abookire, Janet Duffy-Anderson, Benjamin J. Laurel, Patrick H. Ressler & Lauren A. Rogers

Contents

Supplemental Table

Table S1: Summary results for best models.

Supplemental Figures

Fig. S1: Spatial domain for GODAS temperature queries.

Fig. S2: Bayesian regression model estimates of cod recruitment from seine data.

Fig. S3: Direct temperature effects on field estimates of pollock recruitment.

Table S1: Parameter estimates for best model from each model set: 2.5%, 50%, and 97.5% posterior quantiles. ZI = Zero inflation, SD = standard deviation, SD Smooth = standard deviation of smooth term, DOY: day of year, temp = egg/larval temperature, SSB = spawning stock biomass, FAR = Fraction of Attributable Risk.

Model set	Parameter	2.5%	50%	97.5%
M1	Intercept	3.22	3.85	4.47
M1	ZI Intercept	-3.18	-2.02	-1.21
M1	SD Smooth: DOY	0.08	1.48	7.39
M1	SD Smooth: Temp	1.92	4.48	12.96
M1	SD Smooth: SSB	0.27	2.46	9.13
M1	ZI SD Smooth: DOY	0.26	3.30	11.52
M1	ZI SD Smooth: Temp	2.21	5.11	14.84
M1	ZI SD Smooth: SSB	0.08	1.47	7.33
M1	SD: bay effect	0.57	1.01	1.72
M1	SD: site effect	0.32	0.58	0.91
M1	ZI SD: bay effect	0.05	0.77	2.24
M1	ZI SD: site effect	0.99	1.45	2.19
M2	Intercept	2.65	3.32	3.94
M2	ZI Intercept	-4.13	-2.61	-1.45
M2	SD Smooth: DOY	0.07	1.35	7.40
M2	ZI SD Smooth: DOY	0.84	5.47	17.08
M2	SD: bay effect	0.57	1.04	1.78
M2	SD: site effect	0.45	0.71	1.07
M2	ZI SD: bay effect	0.27	1.44	3.36
M2	ZI SD: site effect	1.16	1.71	2.53
M3	Intercept	-1.08	-0.84	-0.58
M3	Seine	0.18	0.35	0.52
M4	Intercept	-0.14	0.07	0.28
M4	SD Smooth: FAR	0.15	2.13	6.98
M5	Intercept	-0.22	0.00	0.23
M5	SD Smooth: FAR	0.79	2.75	8.03

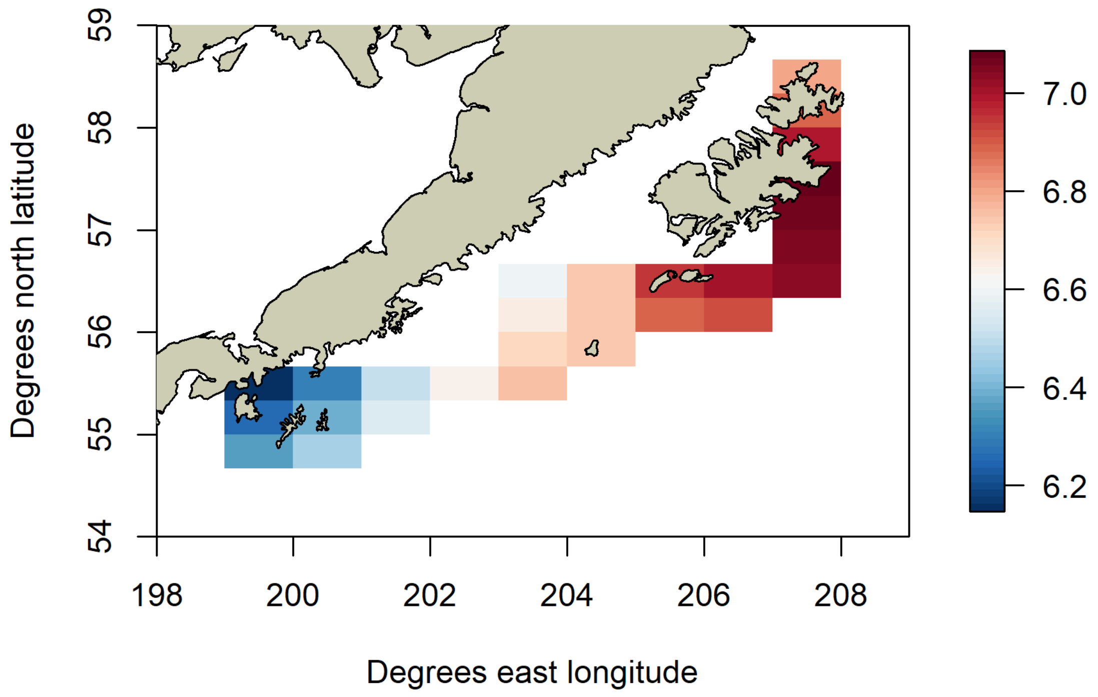

Fig. S1: Query area for GODAS temperature data. Example shows mean May-June 25 m temperature (°C). Map created in R 4.0.2, http://www.r-project.org/.

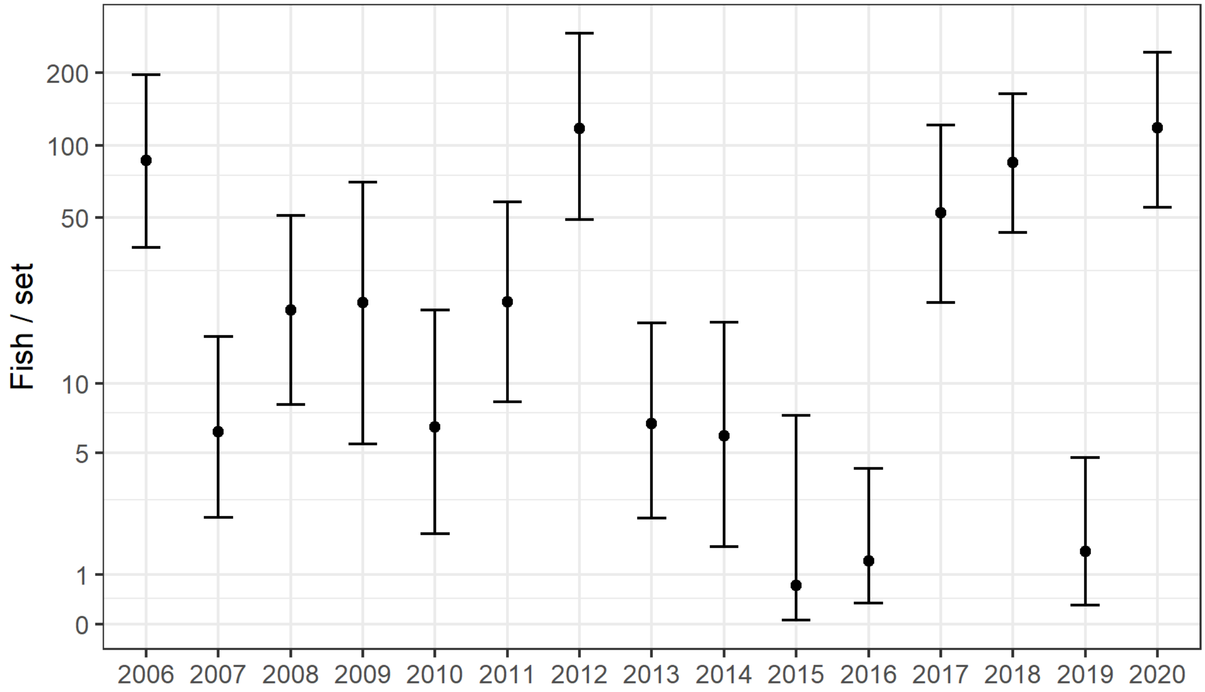

Fig. S2: Beach seine observations of cod recruitment. Bayesian zero-inflated negative binomial estimates of annual age-0 abundance with 95% CIs, from the best model in model set M2 (Tables S1, S2).

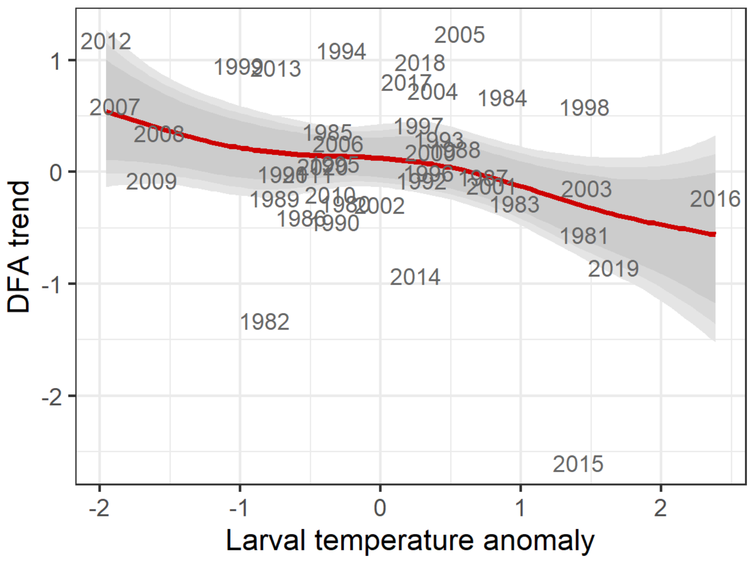

Fig. S3: Direct temperature effects on field estimates of pollock recruitment. Bayesian regression model estimates of DFA trend estimating annual age-0 abundance with 80% / 90% / 95% CIs, from the best model invoking direct temperature effects, rather than FAR estimates, in model set M4 (Table 1).
